# Data-Efficient Equivariant NNPs Enable DFT-Accurate Simulations and Implicit Solvation Free Energies

**DOI:** 10.1021/acs.jpcb.5c05891

**Published:** 2025-11-28

**Authors:** Esma Mutlu, Selonou G. Kankinou, Omer Tayfuroglu, Abdulkadir Kocak

**Affiliations:** † Department of Chemistry, 52962 Gebze Technical University, Kocaeli 41400, Turkey; ‡ Department of Chemical and Biological Engineering, 52979 Koc University, Istanbul 34450, Turkey

## Abstract

The use of machine learning (ML) potentials has emerged as a powerful approach in computational chemistry, particularly in computer-aided drug design studies. Neural network potentials (NNPs) provide a more physics-informed estimation of binding and solvation events, bridging the accuracy gap between classical force fields and quantum mechanical methods. Current universal neural network potentials (NNPs) have not yet achieved consistent chemical accuracy required for reliable molecular dynamics simulations. Accurate yet data efficient representation of potential energy surfaces and prediction of solvation free energies are essential for large-scale molecular simulations and drug-design workflows. Here, we utilize data efficient E(3)-equivariant graph neural network potentials that are capable of estimating the solvation free energies (SFEs) of small compounds with density functional theory (DFT)-level accuracy and significantly reduced computational cost. Leveraging the data efficiency of equivariant architectures, our models achieve chemical accuracy with a relatively small training data set. We demonstrate that the method is data-efficient in constructing ML potentials. Our focus is on hydration free energy changes of small compounds from the FreeSolv database. We develop and test two distinct NNPs—one for the gas phase and one for an implicit water model—that can be applied in molecular simulations and SFE calculations using implicit solvation. The Solvation Model based on Density (SMD)-based implicit NNP model achieves an accuracy of 89% while offering a substantial computational speed-up compared to its DFT counterpart, which attains 90% accuracy.

## Graphical abstract

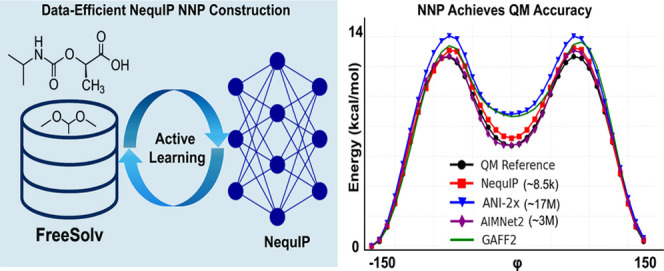

## 1 Introduction

In computer-aided drug design and discovery, achieving the optimal balance between computational cost and accuracy remains a significant challenge when evaluating drug candidates as potential inhibitors. Numerous computational strategies have been developed, each with distinct strengths and limitations. Molecular dynamics (MD) simulations are essential for probing physicochemical and biochemical properties, such as stability, solubility, binding and solvation free energies.^1–35^ MD simulations are particularly important in the accurate and efficient prediction of binding free energy (BFE) changes upon ligand binding to biological targets.^1–36^ However, most MD-based methods, rely on molecular mechanics (MM)-based representations of the system’s multidimensional potential energy surface (PES) through classical force fields (FFs) with empirical parameters. In principle, a PES is best described by quantum mechanical simulations, such as ab initio molecular dynamics (AIMD), yet, even with current computational resources, AIMD simulations of large biomolecular systems remain prohibitively expensive.

Among the many factors influencing BFE calculations, solvation free energy (SFE) plays a particularly critical role. SFE is not only essential for assessing the solubility and bioavailability of drug candidates but is also a key component in most thermodynamic cycles used for BFE estimation. Ligand binding in aqueous media is a complex, multistep process, and one of these steps often involves predicting the change in SFE upon binding. As a result, accurate prediction of ligand SFEs remains in high demand, even when the primary objective is BFE calculation. Like BFE estimations, SFE calculations are subject to the same limitations inherent in classical force fields, further motivating the search for improved approaches.

Over the past decade, machine learning (ML) has emerged as a transformative tool across scientific disciplines, including computational chemistry, owing to its data-driven nature and adaptability. In particular, neural network potentials (NNPs) show strong promise in delivering quantum mechanical (QM) accuracy at near molecular mechanics (MM) computational cost, effectively bridging the accuracy–performance gap.

The most prominent examples of NNPs are ANI and AIMNET.^36–41^ These models have been successfully applied to energy minimization and low-lying minimum searches using various gradient-based minimization algorithms,^42,43^ binding^44,45^ and solvation free energies,^31,33,34,46,47^ scoring functions in molecular docking,^48^ conformer generation,^49^ as well as hybrid ML-MM simulations,^50–52^ analogous to the QM/MM framework. Recently Isayev and co-workers^39^ introduced AIMNet2, which extends coverage to 14 elements in both neutral and charged states and incorporates long-range corrections, thereby enabling a broad range of new applications. Despite this progress, still fully ML-based MD simulations of large chemical or biomolecular systems, to the best of our knowledge, have yet to be demonstrated. Indeed, a few studies suggest that current NNPs are not yet ready for such applications.^44,53^

A common limitation of NNPs in bulk-phase MD is the decline in accuracy as system size grows-a problem also observed in classical force fields (FFs). To achieve broader chemical coverage, whether with a classical FF or an NNP, generalization is required. However, this generalization can increase errors beyond the threshold of chemical accuracy (≈1 kcal/mol), ultimately compromising the correct description of physically governed processes. This raises a key question: should the field aim for a single, broadly applicable NNP that covers large chemical spaces but sacrifices chemical accuracy, or focus on purpose-specific NNPs that are restricted in scope but retain accuracy within chemical limits?

Recent developments in equivariant neural network potentials, such as Neural Equivariant Interatomic Potential (NequIP) offer promising solutions to several of these limitations.^54,55^ By explicitly incorporating the symmetries of three-dimensional space, these architectures can learn interatomic potentials more efficiently and generalize better across configurations. As a result, equivariant NNPs often require significantly less training data to achieve chemical accuracy, exhibit smoother and more physically consistent potential energy surfaces (PES), and demonstrate improved transferability to systems outside their original training set. These features position equivariant NNPs as strong candidates for applications that demand both high accuracy and computational efficiency, including large-scale molecular simulations and free energy calculations.^55^

In this study, we employ NequIP architecture to construct data-efficient, purpose-specific NNPs capable of reproducing DFT-level energies for small organic compounds from the FreeSolv database, both in the gas phase and in implicit aqueous solvation (SMD model). Leveraging the inherent data efficiency of the equivariant architecture, these models are trained on a relatively small data set yet achieve chemical accuracy. We demonstrate that they can be applied directly in MD simulations and used for implicit SFE calculations, offering a fast, accurate, and data-efficient alternative to conventional QM-based approaches.

## 2 Computational Methods

Custom Python scripts, along with Openbabel^56^ and atomic simulation environment (ASE),^57^ were extensively used for root-mean-square distance (rmsd) calculations, file conversions, conformer generations, the construction and evaluation NNPs. Visualization and structural analyses were performed using PyMOL,^58^ GaussView 6,^59^ ChemOffice,^60^ while graphical representations were generated using Matplotlib Python library.^61^

### 2.1 Model Training

#### 2.1.1 Workflow

The potential energy surface (PES) was modeled using NequIP, which represents both bonded and nonbonded interactions. NequIP is well-known for its ability to accurately capture bonded interactions with high data efficiency, establishing a benchmark in the field.^62^ By learning atomic properties directly within an equivariant framework, it enables training with significantly less data than conventional models.^63^ For these reasons, NequIP was selected as the neural network architecture for NNP construction in this study.

The overall workflow used to train the NNP is summarized in Scheme 1. Briefly, the data set was generated by producing nonequilibrium geometries of molecules from the FreeSolv database, performing DFT calculations, and applying active learning cycles, ultimately yielding the final NNP.

**Scheme 1:**
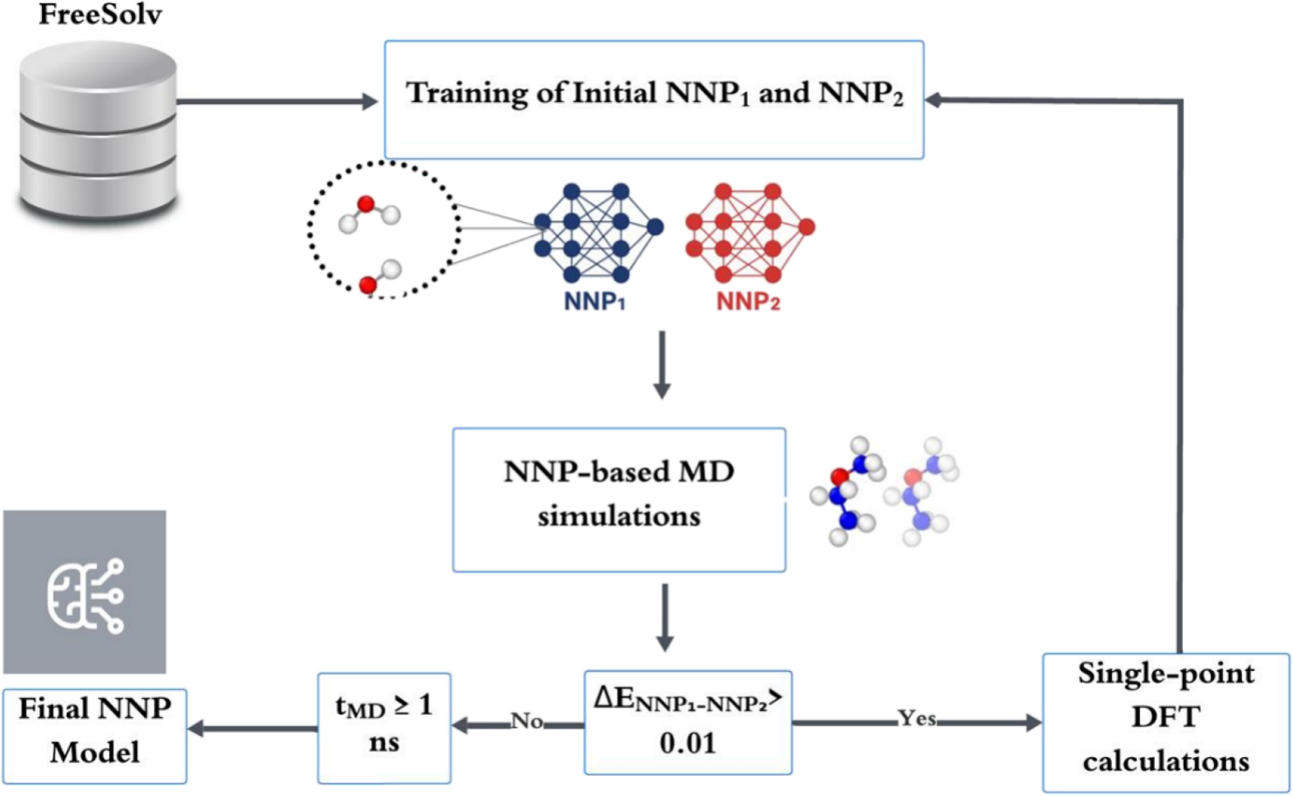
Computational Workflow of NequIP-Based NNP Construction

#### 2.1.2 Initial Data Set

FreeSolv database introduced by Mobley and Guthrie.^64^ contains 645 small molecules with experimental hydration free energies, along with calculated by Bennet-Acceptance Ratio (BAR) method using classical MD. Since data for other elements are sparse, we selected only 407 molecules composed of C, H, O, and N atoms. Approximately 3500 nonequilibrium conformers were generated from these molecules following established protocols.^65–67^ Each molecule was placed in a cubic cell and uniformly scaled from 0.96 to 1.10 Å in increments of ∼0.02 Å, followed by random atomic displacements of up to 0.16 Å to enhance structural diversity. These perturbations produced a diverse ensemble of nonequilibrium configurations that formed the initial data set for NNP training and active learning. For each structure, single-point energies were computed in the gas phase using Gaussian 16 at the ωB97XD/6–311++G­(3df,3pd) level, consistent with the functional family employed in ANI and AIMNet models to enable direct benchmarking. All structural and energetic information from this data set was converted into the ASE-readable format.

Previous studies have reported that NNPs can be developed more effectively using cohesive energies rather than total energies.^65^ Cohesive energy is defined as the sum of all interatomic interactions, excluding atomization energies

1 $$E_{cohesive}=E_{total}-\sum_{i=1}^{atoms}E_{i}$$

where *E*_total_ is the molecular potential (SCF energy) and *E*_*i*_ are the ground-state electronic energies of isolated atoms. We tested this strategy and observed that cohesive-energy–based training yielded markedly lower training and validation losses, along with at least a 5-fold reduction in mean absolute error (MAE) for both per-atom energies and forces (Table S1). To improve predictive performance and physical interpretability, we therefore used cohesive energies^65^ as the training target rather than total energy. The atomization energies are listed in Table S2.

#### 2.1.3 Active Learning

We followed a protocol similar to our previous studies,^66,67^ consisting of two iterative processes to ensure that configurations better represented relevant regions of the PES.

1. **Model disagreement.** Two NNP models (NNP_1_ and NNP_2_) were trained on the same initial data set but initialized with different random weights. Discrepancies between their energy predictions (*E*_NNP1_ and *E*_NNP2_) were used to identify underrepresented regions of the PES. Structures with the largest discrepancies were prioritized for DFT single-point calculations and added to the training data set.
2. **MD-driven sampling.** Molecular dynamics simulations were carried out with NNP_1_ in ASE under the *NVT* ensemble for 1 ns, at temperatures ranging from 100–300 K, with a time step of 0.5 fs. For each generated geometry, the energy was recalculated using NNP_2_. Configurations showing energy differences above a predefined threshold were selected for DFT-based calculations and incorporated into the data set.

This active learning cycle was iteratively repeated until energy differences for newly generated configurations fell below the threshold.

#### 2.1.4 Final Data Set

After the iterative active learning process involving MD-driven sampling and model disagreement, we obtained the final NNP models for the gas phase and SMD water environments. The final data set consisted of ∼8500 geometries across 407 molecules, verified through stable 1 ns MD simulations and low RMSE values in both energies and forces.

#### 2.1.5 Implicit Solvation Model

In addition to gas-phase NNPs, an SMD-based NNP model was also constructed to represent an aqueous solvent environment.

### 2.2 Molecular Dynamics Simulations

#### 2.2.1 Ab Initio Molecular Dynamics Simulations

AIMD simulations were performed using the CP2K package (Quickstep module) at the ωB97X-D/6–31G­(d) level, with all-electron Gaussian basis sets obtained from the EMSL library. A plane-wave cutoff of 400 Ry and a relative cutoff of 60 Ry were employed. The self-consistent field (SCF) procedure used orbital transformation with DIIS and full preconditioning. An inner-loop convergence criterion of 1 × 10^–8^ was applied; if convergence was not achieved within 50 iterations, an outer loop with a looser threshold of 1 × 10^–6^ was used.

Prior to MD, each system was geometry-optimized using a conjugate gradient algorithm until the maximum force, displacement, and their root-mean-square values were below 1 × 10^–2^ a.u. The optimized molecules were then placed in cubic simulation boxes of 14.0 Å without periodic boundary conditions. *NVT* simulations were performed at 450 K using a CSVR thermostat (time constant 1 ps), with a time step of 0.5 fs for a total of 200 ps. Atomic coordinates, velocities, and energies were recorded at every step. To ensure consistency with the Gaussian-based reference data used for NNP training and testing, the AIMD trajectory coordinates were recalculated at the ωB97X-D/6–311++G­(3df,3pd) level using Gaussian 16.

#### 2.2.2 Classical MD Simulations

Classical MD simulations were carried out with GROMACS 2025.2.^68^ Ligand parameters were generated with the GAFF2 force field using AmberTools,^69^ assigning AM1-BCC charges.^70^ Because nonperiodic simulations were not supported in this version of GROMACS, each system was placed in a large cubic box of 45.0 Å to minimize self-interactions under periodic boundary conditions.

Simulations were conducted in the *NVT* ensemble at 450 K using a velocity-rescaling thermostat (τ = 2.0 ps), with a time step of 0.5 fs for 200 ps. Long-range electrostatics were treated using the particle mesh Ewald (PME) method (real-space cutoff 1.0 nm, PME order 4, Fourier grid spacing 0.16 nm). van der Waals interactions were truncated at 1.0 nm. Bond lengths were left unconstrained, and the Verlet cutoff scheme was used with neighbor-list updates every 20 steps.

#### 2.2.3 Machine Learning–Based Molecular Dynamics Simulations

ML-based MD simulations were performed using two different neural network potentials. First, GROMACS 2025.2 was compiled with ANI-ML potentials in the QM/MM framework. In this setup, ANI-2x was used as the NNP to describe the entire system, while all other parameters were identical to those in the classical MD protocol. Second, NequIP-based MD simulations were performed with the LAMMPS package^71,72^ in the *NVT* ensemble at 450 K. A Nosé–Hoover thermostat was applied, with a time step of 0.5 fs for a total simulation time of 200 ps. The last 100 ps was used for the analysis.

### 2.3 DFT Calculations

All DFT calculations were performed using Gaussian 16 at the ωB97XD/6–311++G­(3df,3pd) level in either the gas phase or SMD implicit water solvation.^73^ Unless otherwise noted, this level of theory is referred to as “DFT” throughout the manuscript. For potential energy scans, relevant coordinates were defined as redundant internal coordinates, and relaxed scans were carried out by incrementally scanning the selected coordinate while reoptimizing the structure at each step. NNPs were then evaluated against these QM reference scans.

### 2.4 Normal Mode Sampling

To evaluate the predictive performance of the NNPs, conformational ensembles were generated via normal mode sampling for selected compounds from the FreeSolv database (Figure S1). The nearest local minima were taken from the reported 3D structures, which were first optimized in the gas phase, followed by frequency calculations.

A custom Python script was developed to generate conformations from QM frequency outputs. For each vibrational mode, the eigenvector matrix [M] was scaled in both forward and backward directions with a displacement factor of 0.8 Å. Each mode was sampled between −0.8­[M] and +0.8­[M] in increments of ∼0.2 Å, producing 10 conformations per mode. For a molecule with 12 atoms (30 vibrational modes), this corresponds to 300 conformations. This protocol provided broad sampling of the PES while remaining computationally feasible.

The choice of a 0.8 Å displacement ensured coverage of a wide range of QM relative energies, extending above 400 kcal/mol. This sampling allowed us to probe far from equilibrium geometries and better highlight differences in NNP performance. QM relative energies of the sampled conformations were taken as reference values for comparison with NNP predictions.

## 3 Results and Discussion

### 3.1 NNP Construction and Tests

Following the active learning procedure described in the Methods section, we generated ∼8500 structures from an initial set of 407 molecules in the FreeSolv data set. The data set was randomly divided into a training set (80%) and a validation set (20%) for the development of the final NNP.

The model architecture employed a cutoff radius of 6.0 Å, four interaction layers, and spherical harmonic features up to rotation order *l* = 2with parity symmetry enabled. A weighted loss function was applied to emphasize force accuracy, with force and energy terms scaled at a ratio of 10:1. Training was performed with the Adam optimizer, using a learning rate of 0.005 and a batch size of 5. The mean absolute error (MAE) in the loss curves plateaued around epoch 500 for both gas-phase and SMD models, indicating diminishing improvements beyond this point (Figure S2).

Both NNPs (SMD and gas) showed excellent agreement with DFT reference data, achieving a Pearson correlation coefficient of *R* ^2^ = 1.000 on ∼1600 unseen nonequilibrium conformations (Figure S3). The root-mean-square error (RMSE) of predicted energies was 0.0112 eV/atom for the gas-phase model and 0.0048 eV/atom for the SMD model.

To further validate performance, the NNPs were tested in 1 ns NVT-MD simulations (Figure S4). Energy and temperature remained stable within expected fluctuations, confirming physically consistent dynamics. These results demonstrate the accuracy and robustness of the developed NNPs, which are discussed in more detail in the following section.

### 3.2 Assessing the Performance of NNPs

The performance of the developed NNPs was assessed using three complementary approaches—PES scans, normal-mode (NM) sampling, and MD simulations—on nine representative compounds. These molecules were selected to balance computational feasibility with chemical diversity, encompassing systems with distinct bonding motifs, functional groups, and low energy barriers to rigorously evaluate the sensitivity and accuracy of the NNPs.

#### 3.2.1 PES Scans

The first step in assessing the performance of the developed NNPs was to compare their predicted potential energies with DFT reference values for isolated compounds in both equilibrium and nonequilibrium geometries. This comparison is critical for several reasons. First, a reliable NNP should reproduce the potential energy surface (PES) smoothly enough to enable stable molecular dynamics simulations and the extraction of physicochemical properties. Second, the relative energies predicted by the NNP must reflect QM reference values, since Boltzmann statistics governing conformational populations in MD simulations depend strongly on relative energies. Third, energy barriers connecting different conformations should remain within chemical accuracy (≈1 kcal/mol) in order to correctly capture the effects of thermal fluctuations on population distributions. To this end, we performed a series of PES scans for selected compounds.

Figure 1 presents relaxed PES scans of representative compounds, showing DFT reference values alongside ML-predicted energies and GAFF2 results. For each method, energies were reported as relative values with respect to the lowest-energy conformation along the scanned coordinate. RMSE and MAE values were calculated by comparing each method’s relative energies to the QM references across all scanned points. Overall, all methods correctly identified the equilibrium coordinate (bond distance, angle, or dihedral). However, NequIP and AIMNet2 consistently outperformed the other approaches. GAFF2 displayed large deviations from QM data, particularly at distorted bond lengths and angles far from equilibrium, leading to very high RMSE values (Table 1). ANI-2x also showed notable deviations for longer bond distances, whereas NequIP maintained close agreement with QM references. We attribute NequIP’s superior performance to its symmetry-preserving equivariant atomic environment representation.

**Figure 1:**
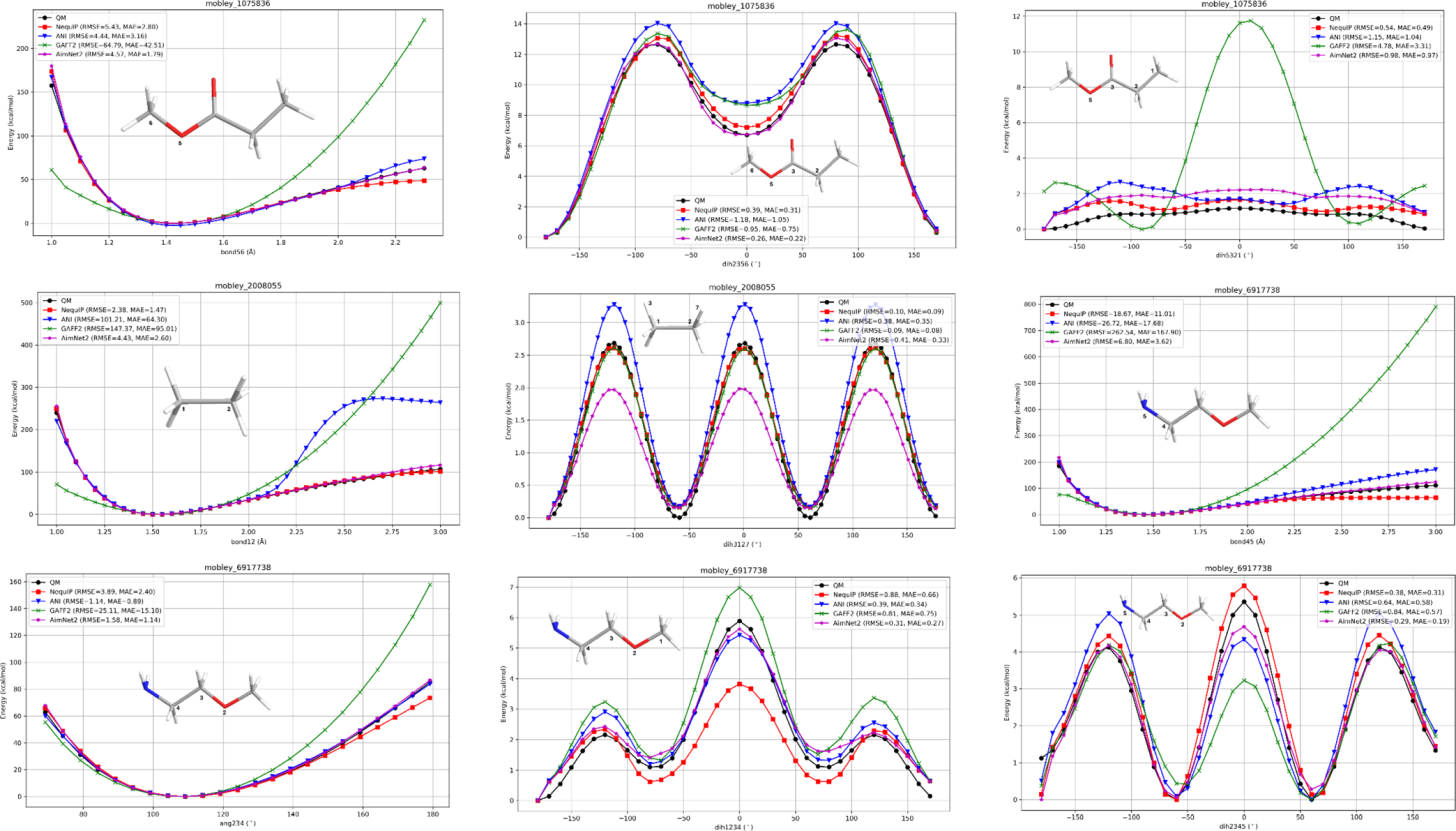
One-dimensional potential surface (PES) scan generated from DFT, NequIP, ANI-2x, GAFF2 and AIMNet2. The atoms in the corresponding scan coordinate are numbered in molecule drawings. The legends show the RMSE of each method with respect to the DFT value.

**Table 1:** RMSE and MAE Statistics of Relaxed QM Scans for Selected Compounds^a^

	ANI-2x		NequIP		GAFF2		AIMNet2	
scanned coordinate	RMSE	MAE	RMSE	MAE	RMSE	MAE	RMSE	MAE
mobley_1075836_bond56	**4.4**	3.2	5.4	2.8	64.8	42.5	4.6	1.8
mobley_1075836_dih2356	1.2	1.0	0.4	0.3	1.0	0.7	**0.3**	0.2
mobley_1075836_dih5321	1.1	1.0	**0.5**	0.5	4.8	3.3	1.0	1.0
mobley_2008055_bond12	101.2	64.3	**2.4**	1.5	147.4	95.0	4.4	2.6
mobley_2008055_dih3127	0.4	0.4	**0.1**	0.1	**0.1**	0.1	0.4	0.3
mobley_6917738_bond45	26.7	17.7	18.7	11.0	262.5	167.9	**6.8**	3.6
mobley_6917738_ang234	**1.1**	0.9	3.9	2.4	25.1	15.1	1.6	1.1
mobley_6917738_dih1234	0.4	0.3	0.9	0.7	0.8	0.7	**0.3**	0.3
mobley_6917738_dih2345	0.6	0.6	0.4	0.3	0.8	0.6	**0.3**	0.2
All	15.2	9.9	3.6	2.2	56.4	36.2	2.2	1.2

a All values are in kcal/mol.

As an illustrative case, we examined methyl propionate by scanning the C6–O5 bond distance (between the methyl group and the propionate oxygen). All methods identified an equilibrium bond distance of 1.4 Å. Both ANI-2x and NequIP accurately reproduced relative energies across the 1.1–2.0 Å range, with RMSE values of 5.4 and 4.4 kcal/mol, respectively. In contrast, GAFF2 deviated substantially outside the 1.3–1.6 Å range, producing an RMSE of 64 kcal/mol. The torsional barrier of the methyl group (∼12 kcal/mol) was reproduced by all methods, although ANI-2x slightly overestimated the barrier. Importantly, the local minimum observed for the trans arrangement of the methyl and carboxyl oxygen atoms (∼7 kcal/mol) was best captured by NequIP.

A second example, ethane, was analyzed by scanning both the C–C bond distance and the rotation around the C–C axis. Both ANI-2x and GAFF2 failed to reproduce QM relative energies when the C–C distance extended beyond 1.3–1.9 Å, resulting in RMSE values as high as 101 and 147 kcal/mol, respectively. In contrast, NequIP maintained excellent agreement with QM references across the entire distance range, yielding an RMSE of only 2.4 kcal/mol. For torsional scans, all methods reproduced the staggered–eclipsed conformations with reasonable accuracy, though ANI-2x showed a slight tendency to overestimate the barrier.

Taken together, these results highlight the robustness of NequIP in reproducing PES features with far fewer training data compared to other models. Its ability to maintain accuracy across both equilibrium and distorted geometries underscores its suitability for MD simulations and free energy calculations.

#### 3.2.2 Normal Mode Sampling

After validating the performance of the NNP in one-dimensional PES scans, we next assessed its predictive accuracy when structures were sampled along normal modes. This step is particularly critical for reliable MD simulations, as molecular structures continuously evolve along these vibrational coordinates during the course of a trajectory.

To benchmark predictive performance across methods, we adopted the cutoff-based RMSE analysis previously applied by König et al.^53^ In this approach, conformations are grouped according to their relative energies with respect to the QM minimum, and RMSE values are computed within specified energy cutoffs. This allows the evaluation of model performance both near equilibrium and in regions far from equilibrium.

For this analysis, 300 conformations were generated for each of seven selected compounds (total 2100 conformers) via normal mode sampling, and their single-point energies were computed at the DFT level. For each molecule, the energy of the lowest-energy conformer was subtracted from all others, yielding QM reference relative energies. Relative energies were then calculated for NequIP, ANI-2x, AIMNet2, and GAFF2 using the same lowest-energy conformer as the reference. RMSE values were obtained by comparing each method against QM data, considering only conformations within the chosen cutoff thresholds. Both cumulative (≤cutoff) and interval-based cutoffs were applied.

Table 2 summarizes the RMSE analysis. Of the 2100 sampled structures, 1660 had QM relative energies within 50 kcal/mol of the minimum. In this near-equilibrium region, all ML-based methods performed reasonably well, with NequIP achieving errors below the threshold of chemical accuracy. As the cutoff energy increased, corresponding to conformations progressively farther from equilibrium, RMSE values grew for all methods, reflecting greater deviation from QM reference data. Remarkably, NequIP maintained errors within chemical accuracy even up to a cumulative cutoff of 300 kcal/mol, highlighting its robustness across a wide energy landscape. Considering the relatively small data set used for training, this level of predictive accuracy underscores the efficiency of NequIP compared to other ML models.

**Table 2:** RMSE Values in Relative Energies of Normal Mode Sampled Conformations for Selected Compounds According to Different QM Relative Energy Cutoffs^a^

= RMSE by cumulative cutoffs = = =						
method	<50	<100	<200	<300	<400	all
NequIP	0.6	0.7	0.8	0.9	1.3	12.6
ANI-2x	1.2	2.1	3.0	3.9	4.9	52.7
AIMNet2	1.0	1.0	1.2	2.1	5.5	55.9
GAFF2	9.0	16.9	32.5	49.5	62.8	105.1
#conformers	1660	1836	1956	2021	2054	2082

= = = RMSE by interval cutoffs (left-inclusive [low, high)) = = =						
method	0–50	50–100	100–200	200–300	300–400	≥400
NequIP	0.6	1.3	1.7	2.7	8.2	123.4
ANI-2x	1.2	5.5	9.2	14.3	23.6	532.2
AIMNet2	1.0	1.5	2.4	8.2	42.8	571.9
GAFF2	9.0	46.4	115.7	217.1	303.2	728.3
#conformers	1660	176	120	65	33	28

a All values except for #conformers (which are number of conformers) are relative energies in kcal/mol.

#### 3.2.3 Molecular Dynamics Simulations of Selected Compounds

As a final validation of our NNP, we examined the energetics along MD trajectories. For this purpose, ab initio MD (AIMD) simulations were performed with CP2K at the ωB97XD/6–31G­(d) level for selected compounds. The resulting MD frames were extracted and recalculated at the higher ωB97XD/6–311++G­(3df,3pd) level, as well as with the trained NNPs. In parallel, ML-based MD (MLMD) simulations were conducted with the NNPs, and the corresponding frames were evaluated with DFT.

For clarity, we use the notation X@Y, where X denotes the energy evaluation method and Y indicates the simulation from which the trajectory was obtained. For example, ML@AIMD represents ML-predicted energies evaluated on AIMD-generated trajectories.

Figure 2 illustrates the potential energy profiles of representative compounds throughout AIMD and MLMD simulations. All energies are reported relative to the initial QM reference energy. Table 3 summarizes statistical analyses of the first 1000 frames for each system. Importantly, the RMSE values for all molecules remained within chemical accuracy, demonstrating that NequIP provides physically consistent energetics along MD trajectories. These results confirm the robustness of NequIP for use in molecular dynamics simulations and support its suitability for free energy applications. The ability to reproduce AIMD-level energetics with such a small training data set further emphasizes the data efficiency of the approach.

**Figure 2:**
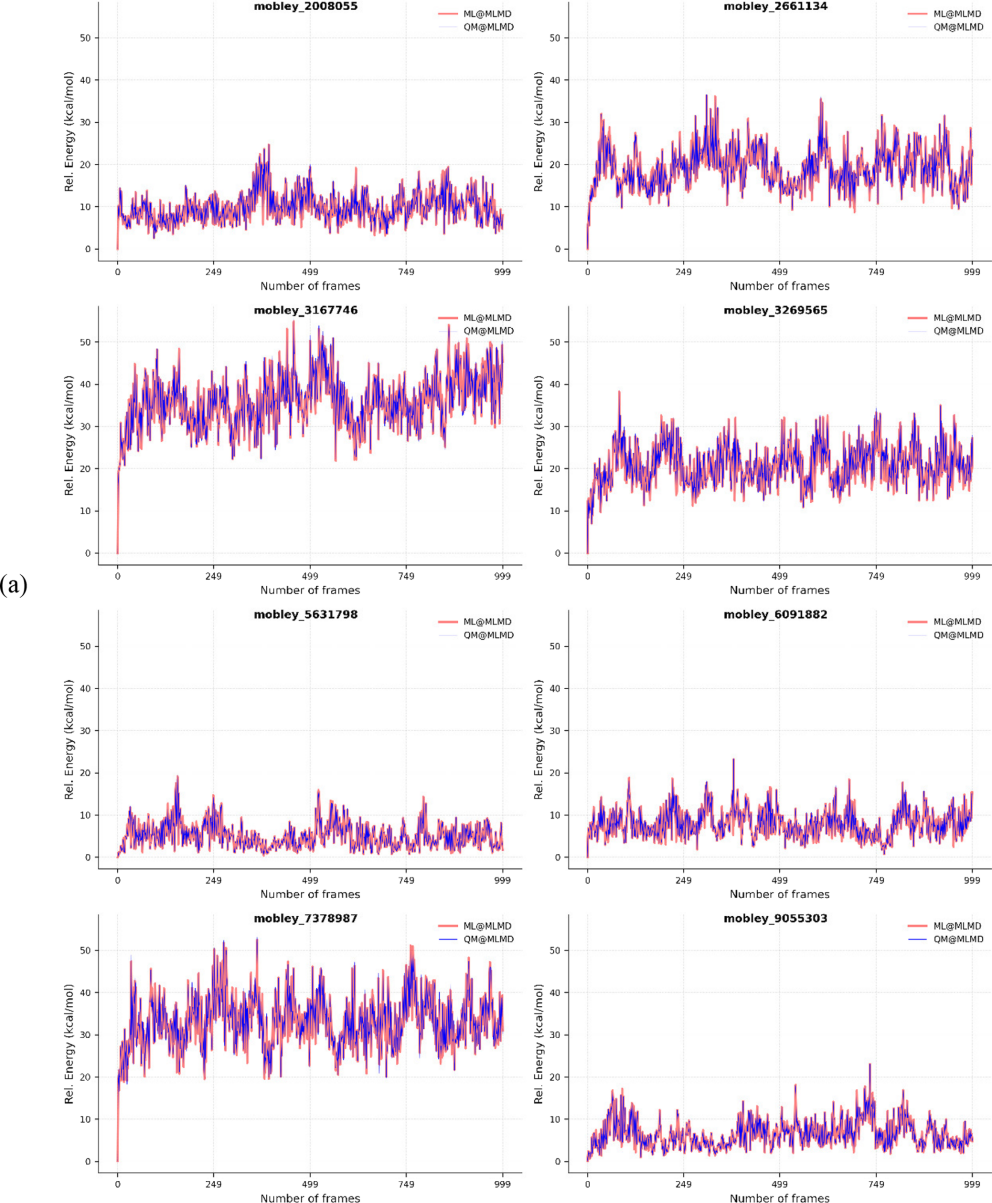

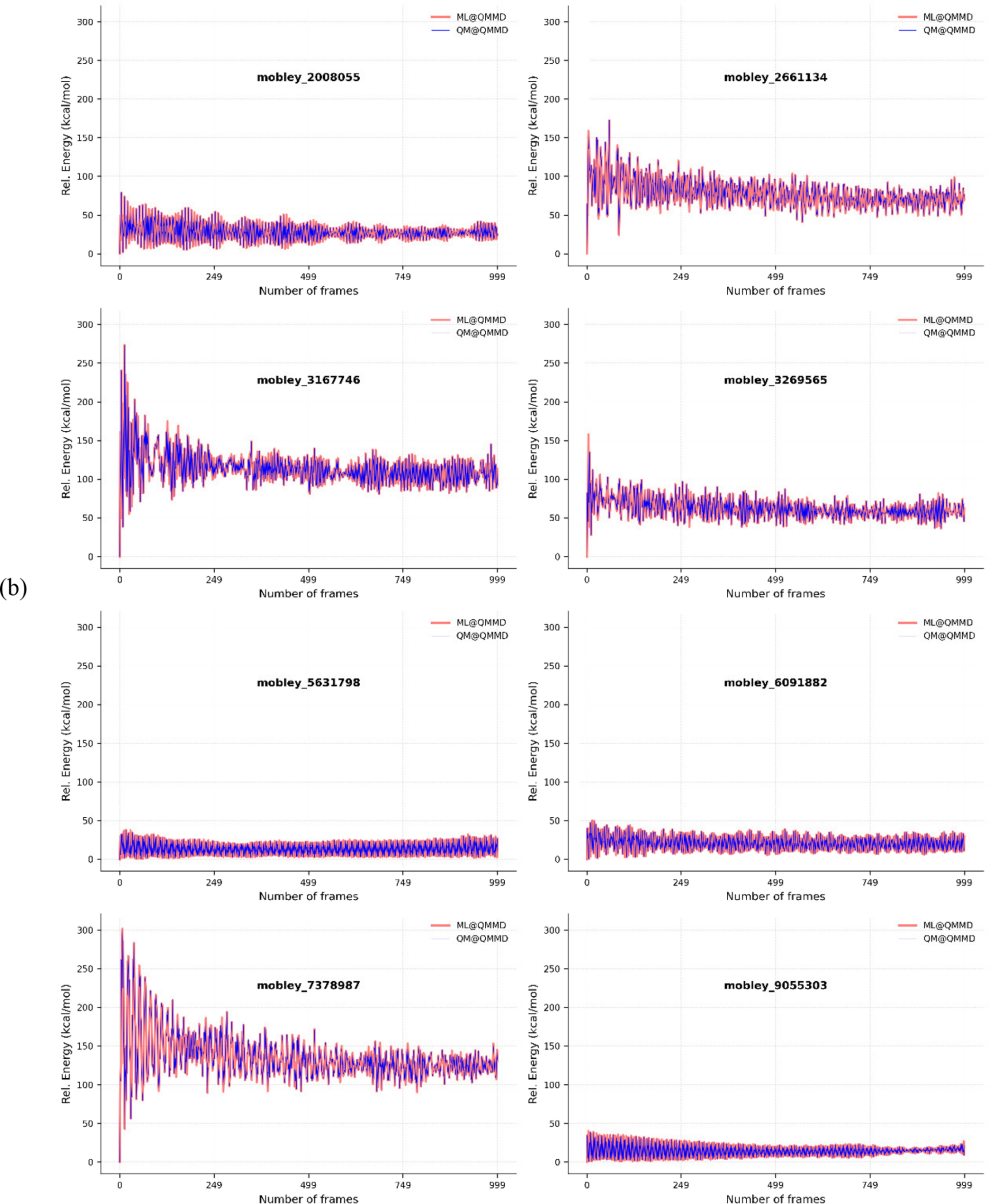
Relative energy change throughout MD simulations calculated on (a) MLMD and (b) AIMD trajectories.

**Table 3:** Statistics for the Relative Energies Calculated Using Different Methods for Selected Compounds^a^

	QM@NequIPMD		NequIP@AIMD	
molecule id	RMSE	MAE	RMSE	MAE
mobley_2661134	0.23	0.18	0.59	0.54
mobley_6911232	0.34	0.24	0.09	0.07
mobley_6091882	0.2	0.14	0.1	0.08
mobley_2008055	0.16	0.1	0.06	0.05
mobley_9055303	0.03	0.02	0.05	0.04
mobley_5631798	0.12	0.06	0.27	0.2
mobley_3269565	0.37	0.27	0.94	0.73
mobley_7378987	0.56	0.44	0.41	0.33
mobley_3167746	0.53	0.42	0.59	0.54

a All values are in kcal/mol.

#### 3.2.4 Spectroscopic and Structural Validation

We further analyzed the MD trajectories by computing vibrational density of states (VDOS) to assess the reliability of the simulations. To ensure sufficient sampling of vibrational populations, simulations were performed at 450 K for 200 ps with a time step of 0.5 fs. Velocities were saved every 2 steps (1 fs), and the last 100 ps of each trajectory were used for VDOS analysis. VDOS spectra were obtained from the Fourier transforms of the mass-weighted velocity autocorrelation functions using custom Python scripts.

Figure 3 compares VDOS obtained from NequIP, ANI-2x, and GAFF2 based simulations with QM harmonic and anharmonic frequencies of fundamental vibrations, as well as experimental gas-phase FTIR spectra retrieved from the NIST Chemistry WebBook (https://webbook.nist.gov/chemistry/). For consistency, QM harmonic and anharmonic spectra were fitted with Lorentzian functions at a resolution of 20 cm^–1^, and the same smoothing procedure was applied to all VDOS spectra.

**Figure 3:**
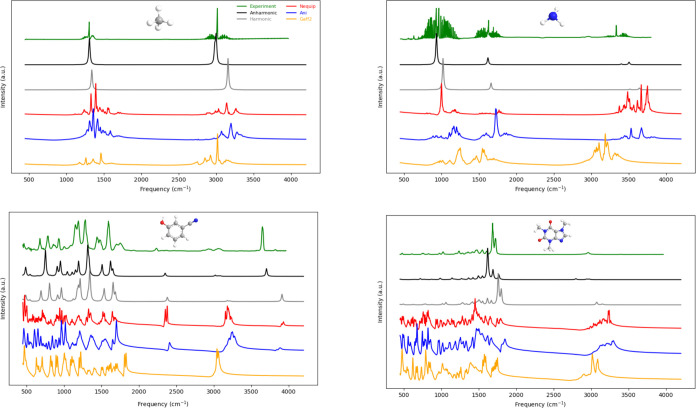
Vibrational spectra obtained from QM harmonic and anharmonic (only fundamental bands) calculations and VDOS analysis of MD simulations compared to experimental spectra for selected compounds.

The results show that all methods successfully reproduced the high-frequency C–H stretching vibrations observed experimentally near 3000 cm^–1^. QM anharmonic calculations almost perfectly captured these motions. Interestingly, although no constraints were applied during the simulations, GAFF2 yielded frequencies closer to the experimental and anharmonic values in this region, whereas ANI-2x and NequIP more closely followed the harmonic predictions. For the O–H stretching vibrations (∼3600 cm^–1^), GAFF2 failed to reproduce the experimental band, while ANI-2x and NequIP showed performance comparable to harmonic QM calculations.

In addition to high-frequency stretching modes, lower-frequency vibrations in the 1000–2500 cm^–1^ region, primarily due to bending motions, were also well reproduced by ML-based simulations. Because anharmonicity is relatively small in this region, experimental, anharmonic, harmonic, and VDOS spectra aligned closely.

Overall, the VDOS analysis supports the reliability of NequIP and ANI-2x based MD simulations in reproducing vibrational features of isolated compounds, while also highlighting specific strengths and limitations of classical and ML-based force fields in capturing vibrational spectra.

We also analyzed the radial distribution functions (RDFs) of selected compounds (Figure S5). The RDF profiles showed good agreement across all methods, with no significant differences observed. This consistency is expected, as equilibrium conformations are well captured by all approaches, in line with the results from the PES scans.

### 3.3 SMD Solvent Environment Testing and Analysis

Because we have constructed NNPs using both gas-phase and SMD implicit solvent environments, we could readily evaluate their performance in predicting solvation free energies (SFEs) based on the energy difference between gas and SMD definitions.

Consistent with our previous work,^33^ we observed a strong correlation (*R* = 0.95) between DFT-based gas/SMD energy differences and experimental hydration free energies (Figure 4a). Here, the DFT reference values were defined as

2 $$\Delta QM=E_{smd}^{QM}@{QM}_{smd}-E_{gas}^{QM}@{QM}_{gas}$$

where the notation “@” denotes the environment used for the optimization, and *E*_smd_^QM^ and *E*_gas_^QM^ are the SCF energies at the bottom of the respective wells.

**Figure 4:**
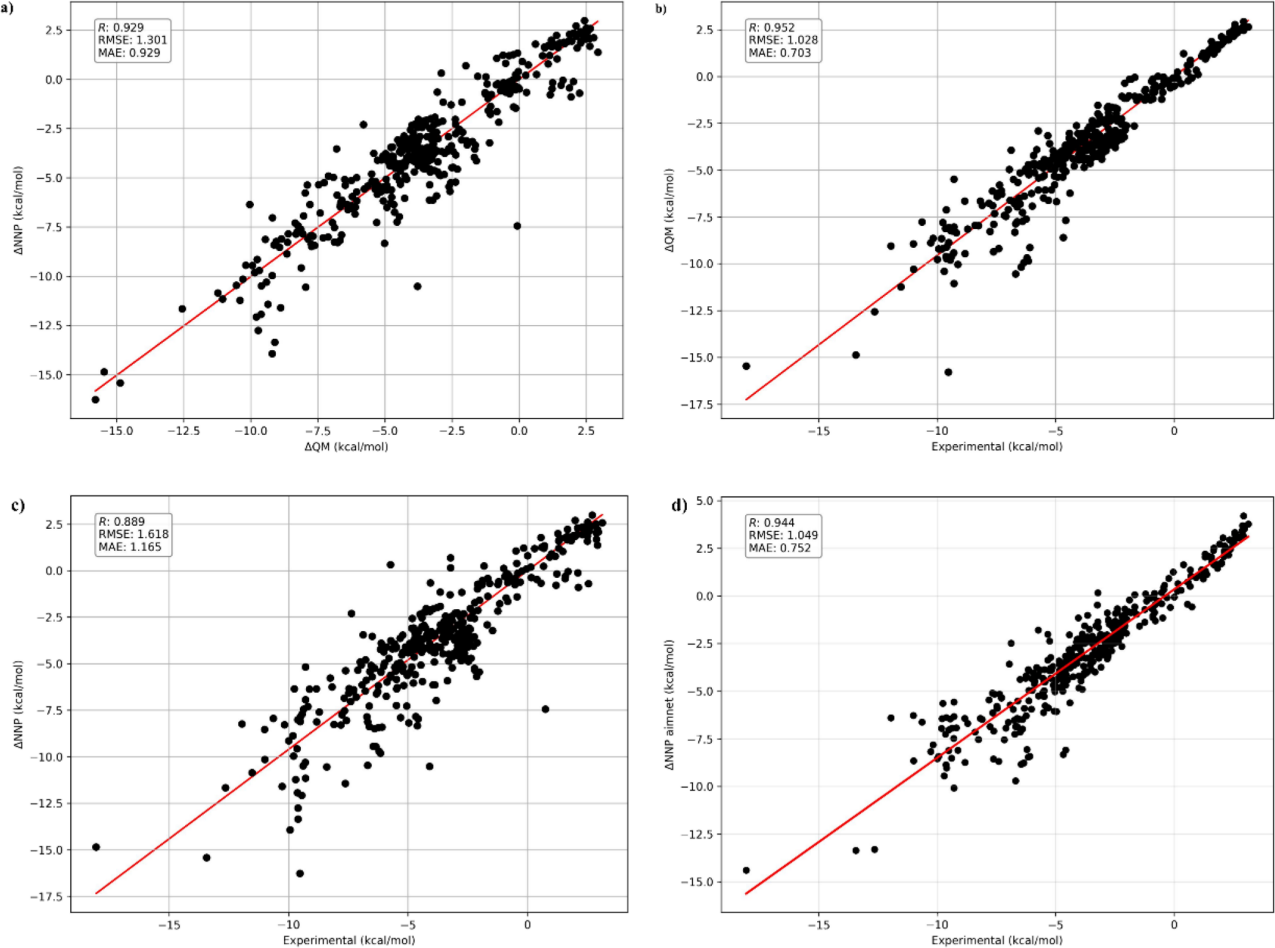
Predictive performance of NNPs the gas phase and SMD implicit water environment and solvation free energies using QM and NNPs (a) NequIP vs QM (b) QM vs experiment (c) NequIP vs experiment and (d) AIMNet vs experiment.

Our NNP based calculations defined as

3 $$\Delta NNP=E_{smd}^{NNP}@{QM}_{smd}-E_{gas}^{NNP}@{QM}_{gas}$$

showed excellent agreement with the QM reference values, yielding a correlation of *R* = 0.93 (Figure 4b). When compared directly with experimental SFEs, the NNP results achieved a correlation of *R* = 0.89 (Figure 4c) This value reflects the combined accuracy of the QM calculations relative to experiment and the NNP predictions relative to QM. For comparison, AIMNet also enabled such calculations, achieving slightly higher correlation with experimental data (*R* = 0.94, Figure 4d).

Although the implicit solvent model involves only ligand atoms (without explicit solvent molecules), the computational cost of performing DFT-level calculations remains high: ∼400 gas-phase and SMD calculations required nearly 3 days on a 112-core machine. By contrast, the same calculations using NNPs were completed within minutes, underscoring the efficiency of ML-based approaches for large-scale SFE predictions.

## 4 Conclusions

NequIP-based neural network potentials achieve chemical accuracy in PES reproduction, MD simulations, and solvation free energy predictions while requiring only a fraction of the data and computational cost of quantum mechanical methods.

We demonstrated that NequIP-based NNPs can accurately represent the potential energy surface (PES) of molecular systems with QM-level fidelity, while remaining computationally efficient and data-light. These NNPs were successfully applied in MLMD simulations of isolated compounds with accuracy comparable to ab initio MD, and in predicting solvation free energies using the implicit SMD model at a fraction of the computational cost of DFT.

Our benchmarks against existing ML potentials highlight an important conclusion: purpose-specific NNPs, trained on relatively small and focused data sets, can achieve chemical accuracy and may be more effective than broadly generalized models trained on millions of data points, which often sacrifice accuracy for transferability. Rather than relying solely on a universal NNP to cover the entire chemical space, targeted models tailored to specific scientific objectives offer a promising and data-efficient alternative. Nevertheless, universal NNPs continue to improve rapidly and may soon achieve chemical accuracy across diverse chemical domains. These models could serve as valuable tools for generating high-quality reference data or specific configurations to train smaller, system-specific NNPs suitable for molecular dynamics simulations—retaining much of the accuracy of DFT while remaining computationally efficient.

## Supplementary Material





## Data Availability

All trained NequIP-based neural network potential (NNP) models, input files, and representative data sets used in this study are freely available at https://github.com/otayfuroglu/deepPotential. The data sets used throughout the manuscript can be accessed via: 10.5281/zenodo.16894903.

## Notes

Additional Supporting Information includes supplementary figures and tables, and raw data of all outputs is provided as Supporting Information (PDF)
Results (XLSX)
