# Supplementary material for: Data-Efficient Equivariant NNPs Enable DFT-Accurate Simulations and Implicit Solvation Free Energies
Source: J Phys Chem B. 2025 Nov 28;129(49):12630–41. doi: 10.1021/acs.jpcb.5c05891 (PMC12703735; doi:10.1021/acs.jpcb.5c05891)
Supplement: Supplementary file 1 [file jp5c05891_si_001.pdf]

Supporting Information

Data-Efficient Equivariant NNPs Enable DFT-Accurate Simulations and Implicit Solvation Free Energies

Esma Mutlu[a], Selonou G. Kankinou[a], Omer Tayfuroglu[a,b] and Abdulkadir Kocak[a*]*

[a] Department of Chemistry, Gebze Technical University, 41400, Kocaeli/Turkey
[b] Department of Chemical and Biological Engineering, Koc University, 34450 Istanbul, Turkey
[*]E-Mail: kocak@gtu.edu.tr; Phone: (262)-605-3083
[*]E-Mail: otayfuroglu@ku.edu.tr

Table S1. The MAE of loss values shows NNP on cohesive energies show better training than total energies. For the comparison epoch value of 300 was used.

	**Training**		**Validation**	
	**Energy/N (eV/atom)**	**Force (eV/Å)**	**Energy/N (eV/atom)**	**Force (eV/Å)**
**$NNP_{total\ energy}$**	0.0509	0.0927	0.0227	0.0955
**$NNP_{cohesive\ energy}$**	0.00354	0.014	0.00354	0.018

Table S2. The atomization energies used in calculating cohesive energies.

**Element**	Energy (eV)
C	-1025.34896263954
O	-2034.97553102427
N	-1479.90402789208
H	-13.5576149854593

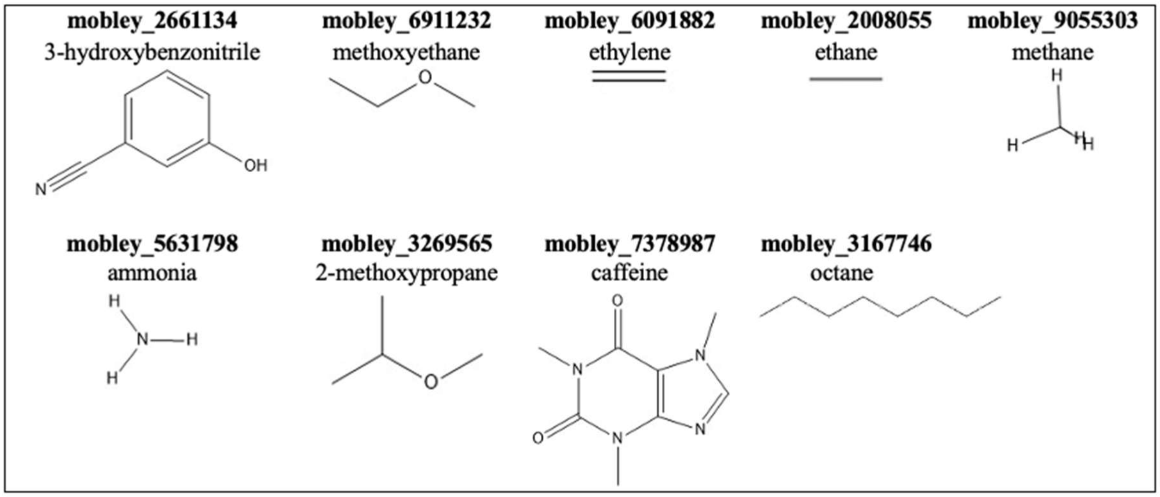

Figure S1. Selected molecules in FreeSolv database used in this study.

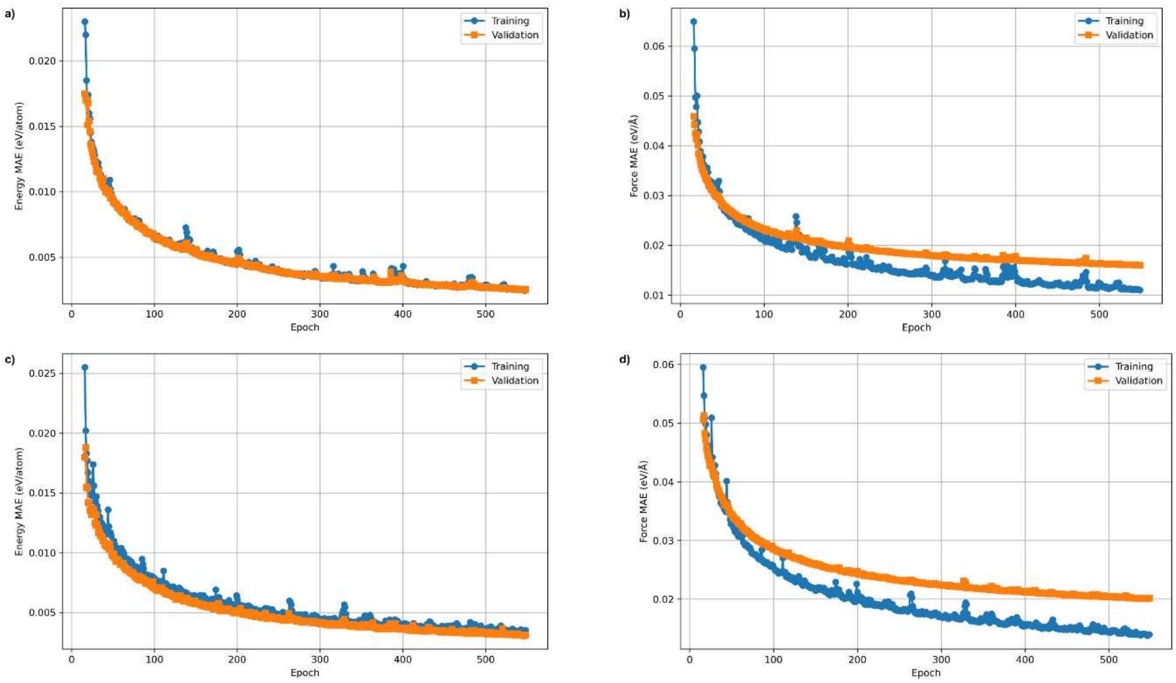

Figure S2. Training and validation MAE of loss values for NNPs on a) gas phase-energy b) gas phase-force, c) SMD implicit water-energy d) SMD implicit water -force.

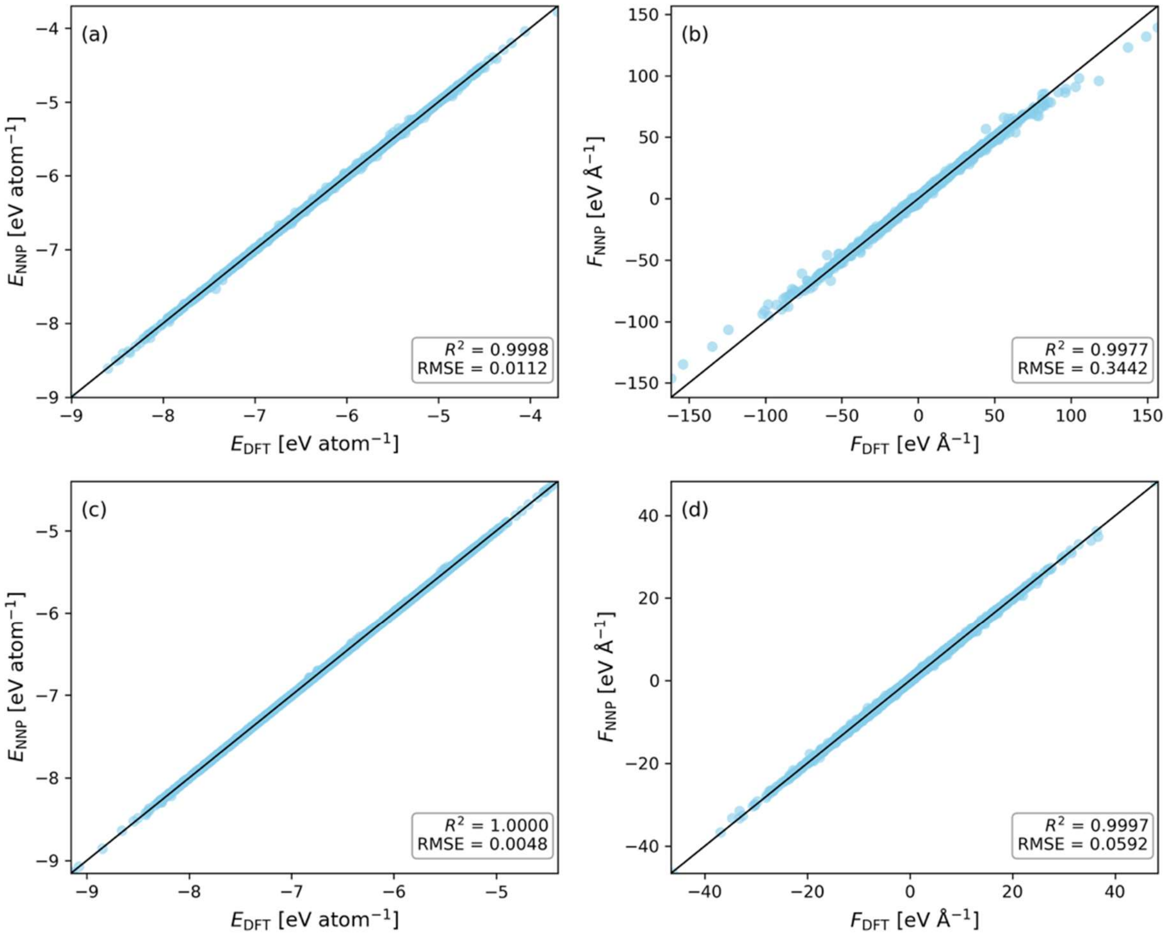

Figure S3. The comparison of DFT vs. NNPs on ~1600 unseen data (non-equilibrium) in the NNP training a) gas phase-energy b) gas phase-force, c) SMD implicit water-energy d) SMD implicit water -force.

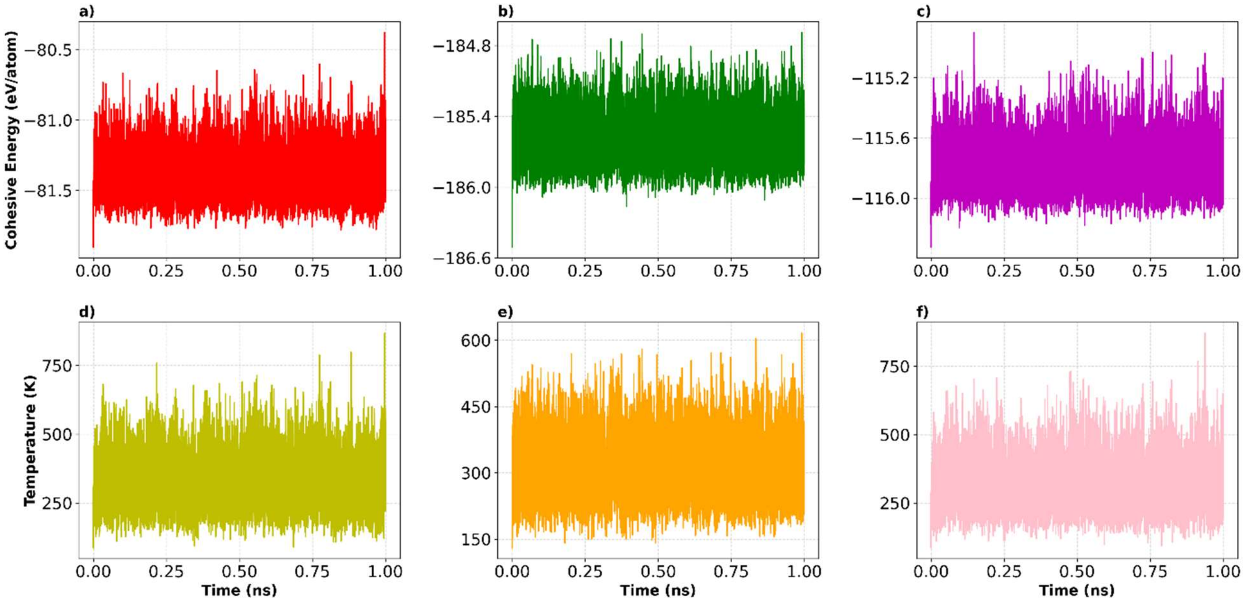

Figure S4. Energy plots (a-c) and temperature plots (d-f) from gas-phase NNP-based NVT-MD simulations at 300 K for randomly selected three molecules.

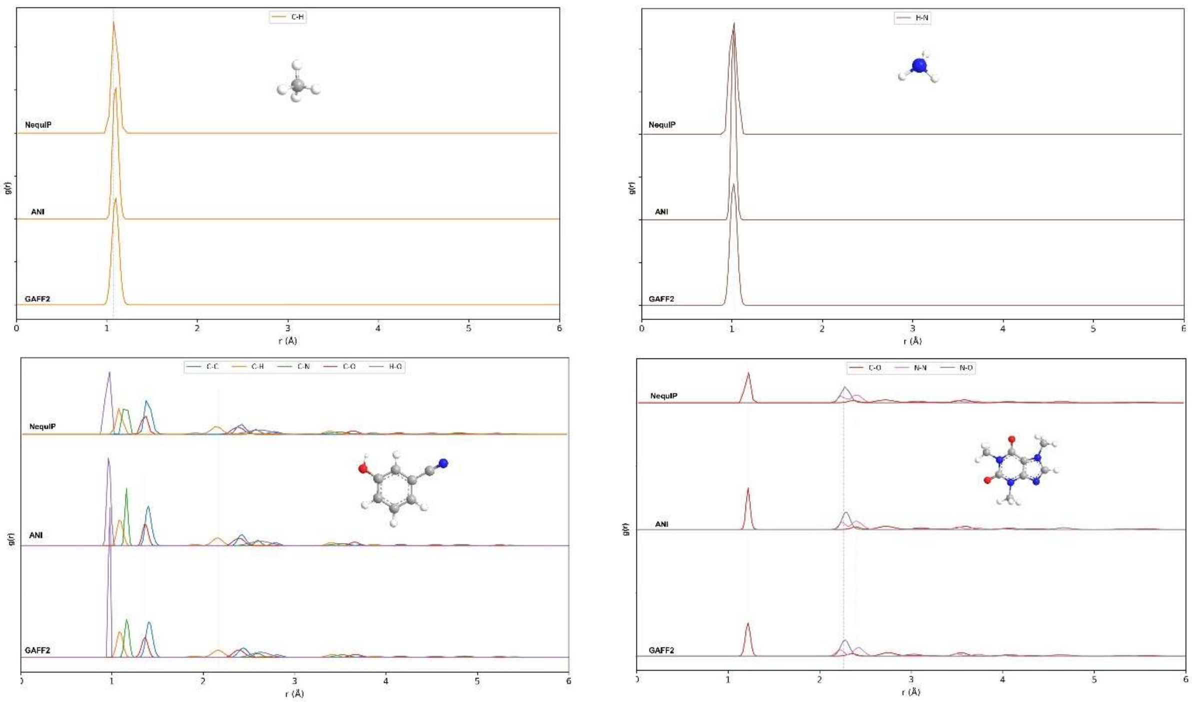

Figure S5. Pair radial distribution functions for selected compounds that are generated by extracting the atom pair distances throughout the MD simulations.
